# Ruptured Hemorrhagic Ovarian Cyst Mimicking Ruptured Ectopic Pregnancy in a Post-Transplant Patient: A Case Report

**DOI:** 10.5070/M5.52390

**Published:** 2026-10-31

**Authors:** Saloni Shah, Kristin Lewis, Andy Hseuh

**Affiliations:** * University of California, Irvine, School of Medicine, Irvine, CA; ^ University of California, Irvine, Department of Emergency Medicine, Orange, CA

## Abstract

The incidence of syncope in the setting of ruptured ectopic pregnancy can vary widely; however, data specifically focusing on syncope as the primary presenting symptom without accompanying abdominal pain or vaginal bleeding are scarce.^1^,^2^ Our 23-year-old female patient, who is post-liver transplant and currently on immunosuppressive therapy, experienced syncope without significant pain or bleeding and presented to the ED with stable vital signs. Initial investigations revealed mild left lower quadrant tenderness, a serum beta-hCG of 95 mIU/mL, and moderate free pelvic fluid with a 28 mm left ovarian “cyst” on transvaginal ultrasound. These findings raised concerns for a ruptured ectopic pregnancy. Diagnostic laparoscopy, however, ultimately revealed a ruptured ovarian hemorrhagic cyst. Subsequent negative urine beta-hCG tests confirmed the initial serum beta-hCG as a false positive likely due to heterophile antibody interference, which is common in immunosuppressed patients. This case represents a rare mimic of a ruptured ectopic pregnancy which can occur in post-transplant patients and a nuanced interpretation of clinical workup.

**Topics:**

Syncope in reproductive-age women, ruptured ovarian cyst, ectopic pregnancy mimics, false-positive beta-hcg, heterophile antibody interference, emergency ultrasonography, diagnostic laparoscopy, immunosuppression and diagnostic challenges, atypical presentations of gynecologic emergencies.

**Figure:**
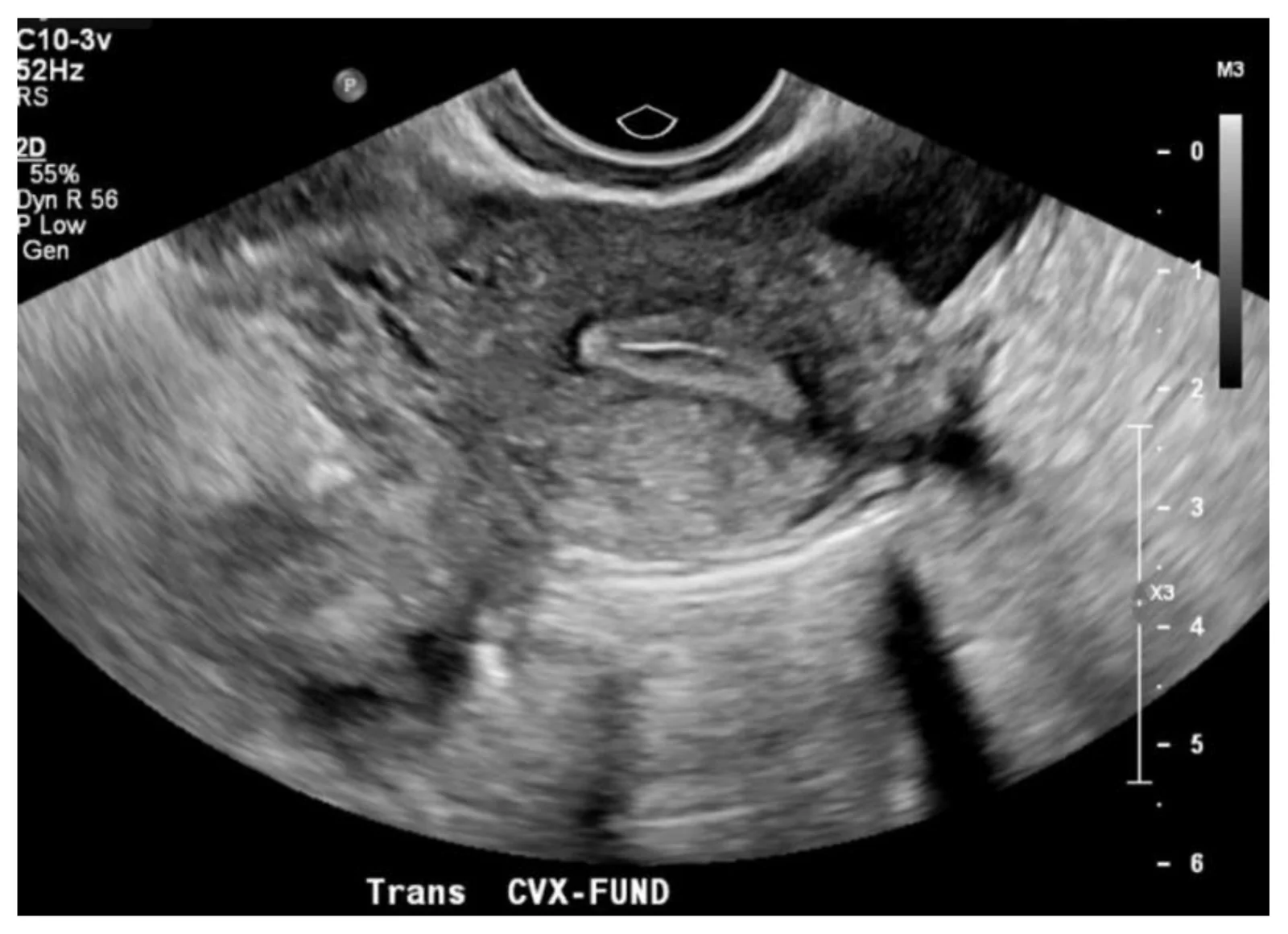


**Figure:**
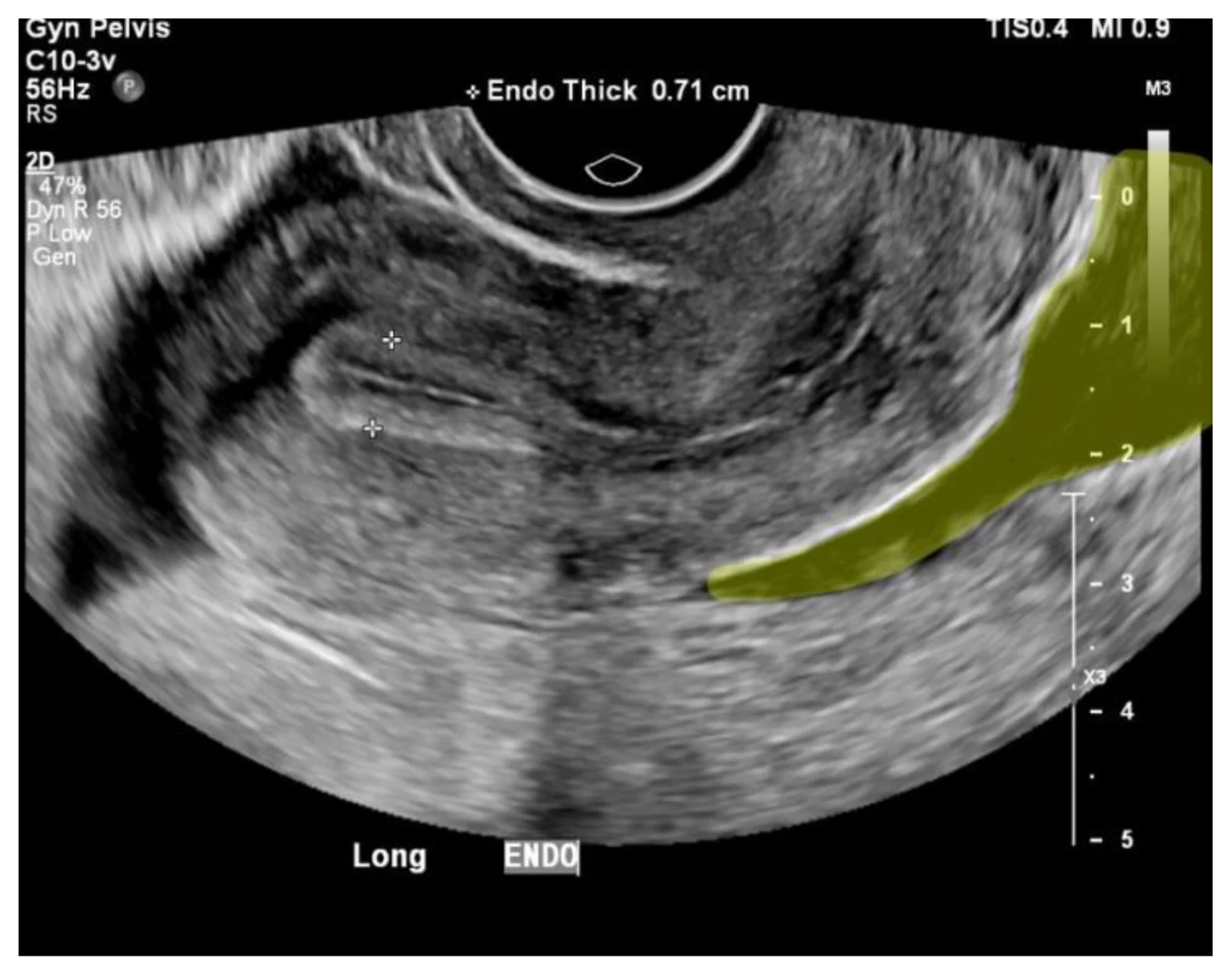


**Figure:**
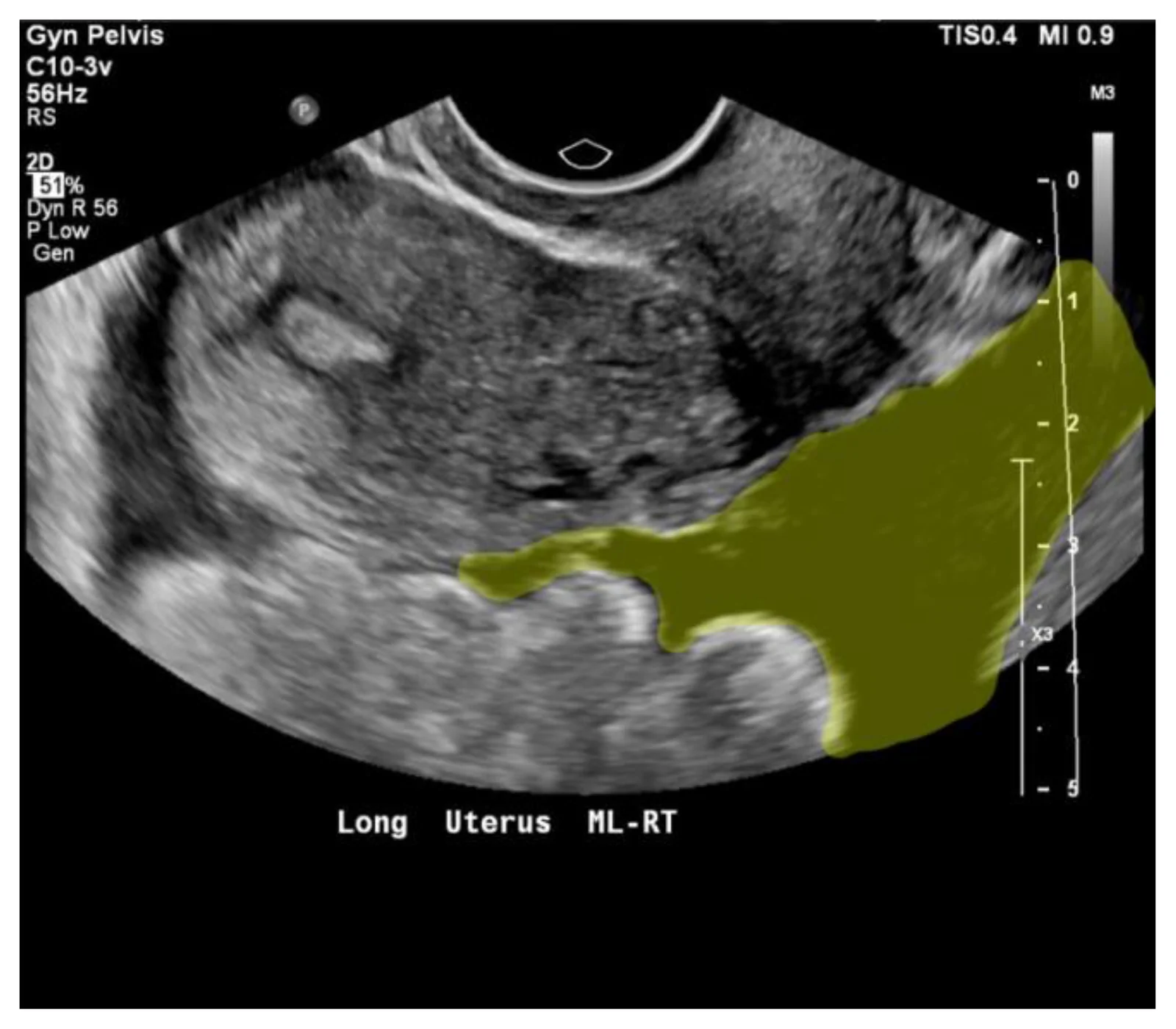


**Figure:**
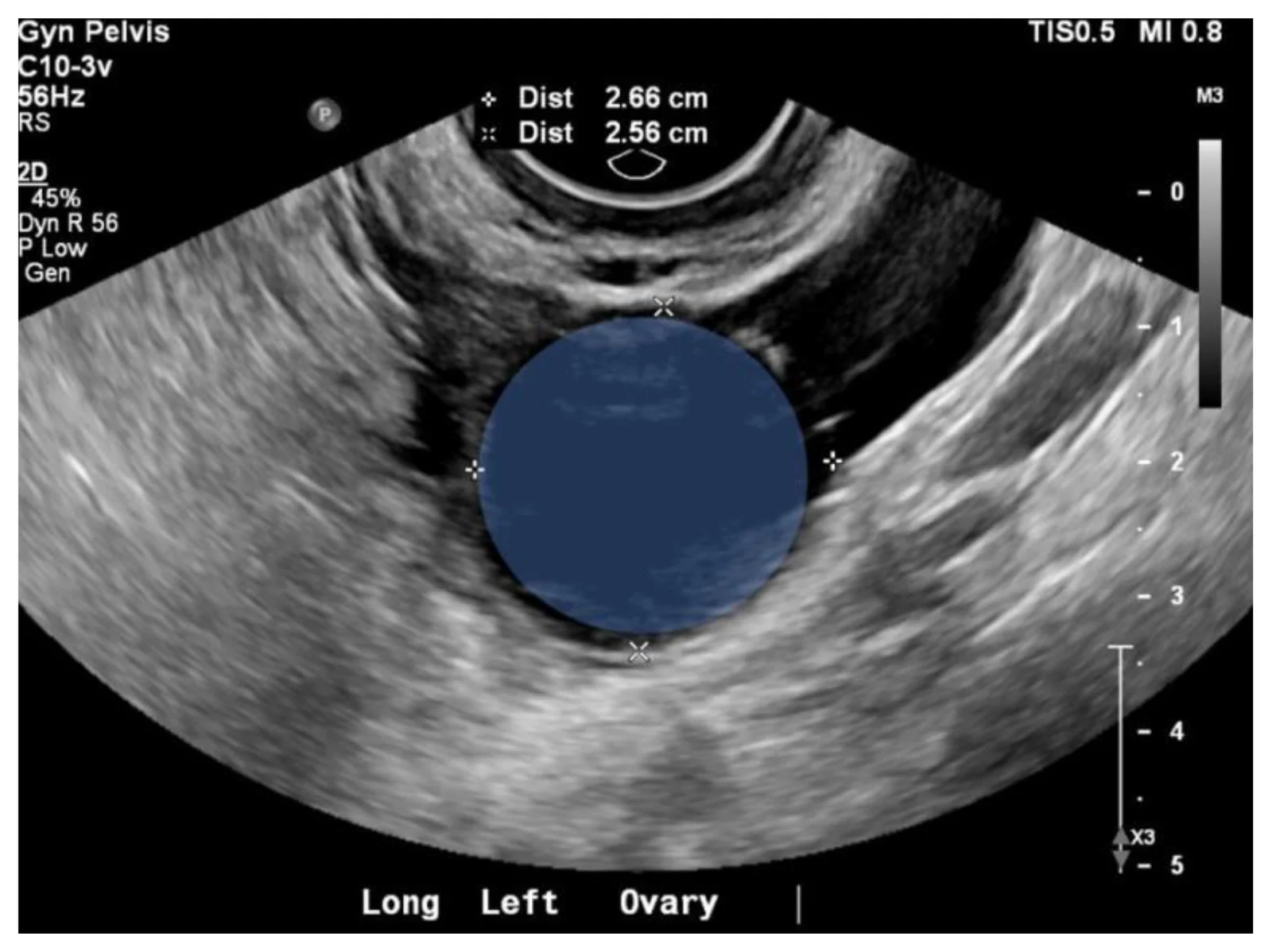


**Figure:**
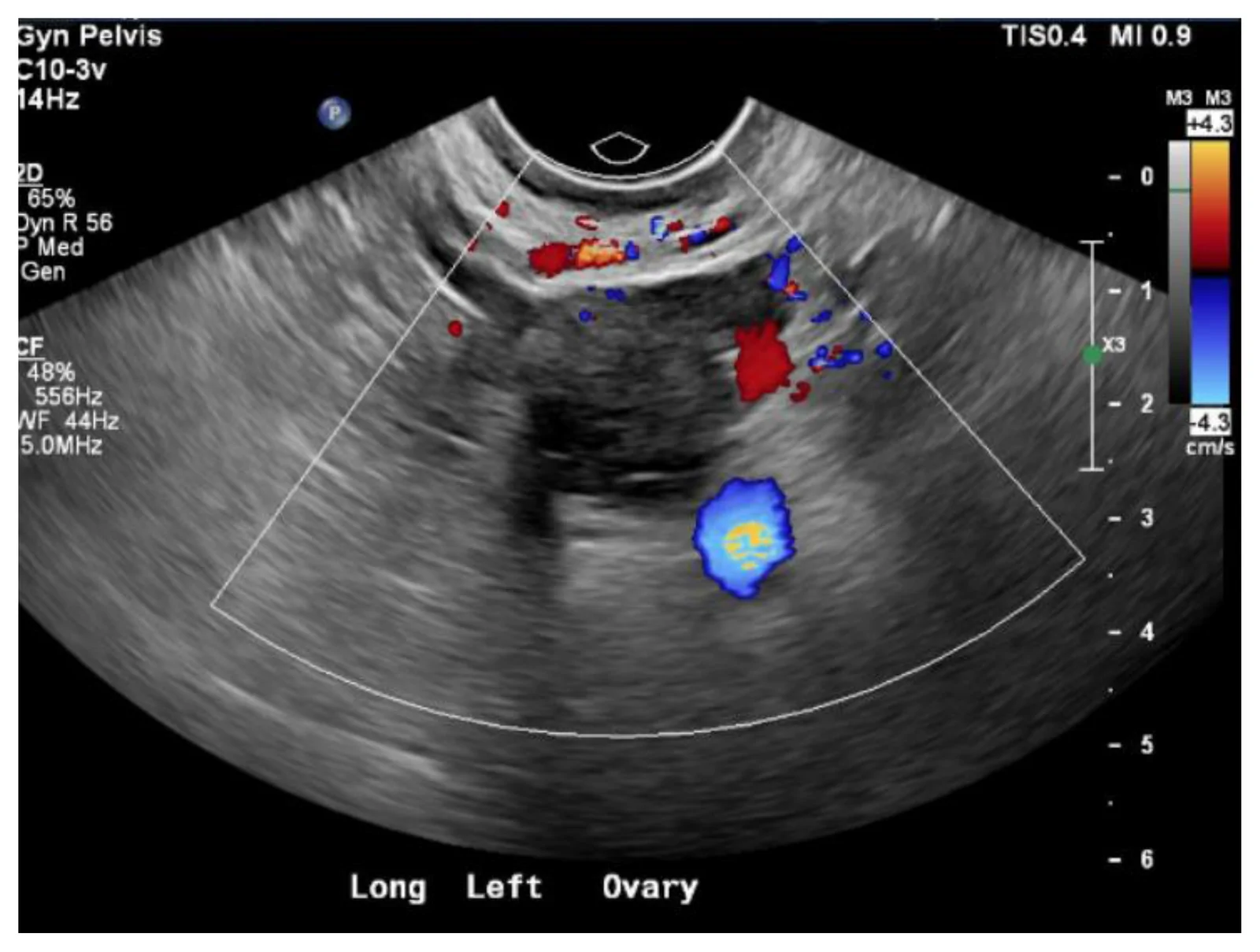


**Figure:**
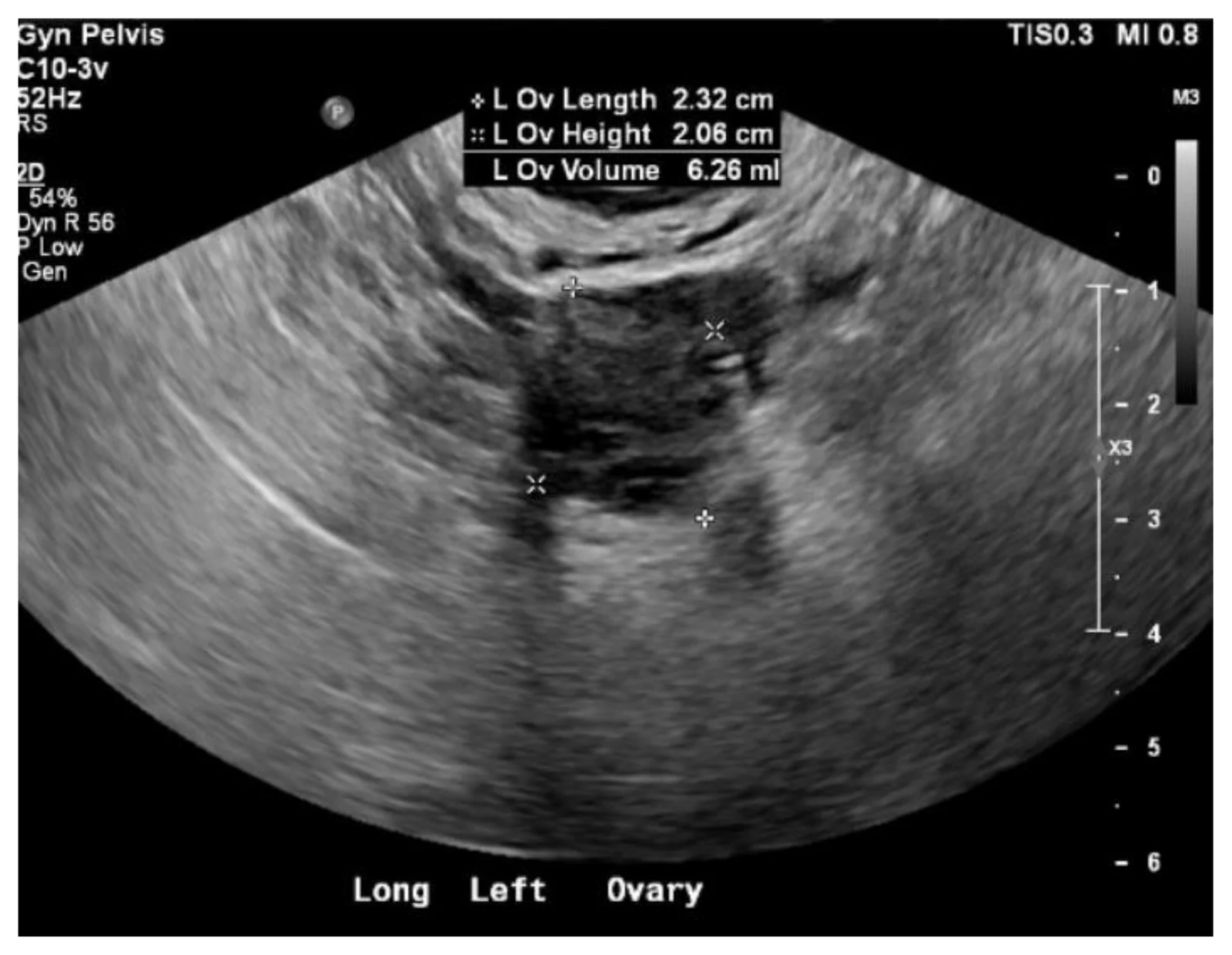


## Brief introduction

Syncope in reproductive-age women can result from various etiologies, ranging from benign to potentially life-threatening. Ruptured ectopic pregnancy is a “can’t miss diagnosis” due to its high morbidity and mortality and is a diagnosis to consider with syncope in this population. Common presenting symptoms of ectopic pregnancy include lower abdominal pain, vaginal bleeding, and dizziness or syncope secondary to internal hemorrhage.^1^,^2^ However, atypical presentations lacking clear abdominal pain or bleeding can pose significant diagnostic challenges for clinicians. In emergency medicine (EM), the diagnostic framework for suspected ectopic pregnancy involves prompt assessment of vital signs, rapid serum beta-hCG testing, and early transvaginal/transabdominal ultrasonography.^1^,^2^,^3^ These initial assessments help to quickly stratify risk and guide management decisions, including urgent surgical intervention when indicated.^3^ Despite established diagnostic algorithms, clinicians can encounter scenarios complicated by false-positive laboratory results and ambiguous ultrasound findings, further complicating clinical decisions and management. This report describes an atypical presentation of syncope ultimately attributed to a ruptured ovarian cyst, initially mimicking ruptured ectopic pregnancy due to a false-positive serum beta-hCG in a rare but growing population of immunosuppressed patients.^4^ This case underscores the need for awareness of atypical mimics of ectopic pregnancy and understanding the nuances of serum beta-hCG for diagnostic purposes.

## Presenting concerns and clinical findings

A 23-year-old female with autoimmune hepatitis status post liver transplant in 2023 and compliant with immunosuppressants (on tacrolimus and prednisone), presented to the emergency department (ED) following a brief syncopal event lasting approximately 10 seconds. Just prior to the episode, the patient experienced tunnel vision, visual darkening with flashes of white light, blurred vision, lightheadedness, and palpitations followed by a brief loss of consciousness lasting approximately 10 seconds. The event was witnessed by her sister, who reported generalized whole-body shaking after the patient was assisted to the ground and denied any associated head trauma. The patient endorsed a similar self-resolving episode several weeks earlier with comparable prodromal symptoms, but without loss of consciousness. She denied chest pain, headache, abdominal pain, nausea, vomiting, dysuria, hematuria, or vaginal bleeding. Her last menstrual period was two weeks prior. She reported being sexually active with male partners and denied use of any birth control method including hormonal contraception. On arrival to the ED, the patient was hemodynamically stable with normal vital signs. Physical examination revealed mild left lower quadrant tenderness without peritoneal signs; no other significant abnormalities such as lower extremity swelling were noted.

## Significant findings

Laboratory results revealed a positive serum beta-hCG level of 95 mIU/mL, with urinalysis showing pyuria and large leukocyte esterase with no prior baseline to compare. The patient’s hemoglobin was within normal limits at 12.6 g/dL, and the rest of her bloodwork as well as her ECG were non-contributory. A formal transvaginal ultrasound was ordered in the setting of the positive beta-hCG levels, which demonstrated a moderate volume of debris-containing free pelvic fluid layering in the posterior cul-de-sac (yellow highlighting), without sonographic evidence of an intrauterine gestational sac or yolk sac. Additionally, the left ovary contained a 2.8 cm cystic structure consistent with a hemorrhagic ovarian cyst (blue highlighting).

## Patient course

Once the patient’s beta-HCG test resulted, she was immediately reassessed. Her vitals remained normal, and her abdominal exam revealed stable mild left lower quadrant tenderness. She was urgently taken for a transvaginal ultrasound. When moderate free pelvic fluid was visualized, OB was consulted. No repeat quantitative or urine pregnancy was obtained prior to the patient undergoing emergent diagnostic laparoscopy, which ultimately revealed a ruptured ovarian hemorrhagic cyst. The patient experienced an otherwise uncomplicated postoperative course and had two subsequent negative urine beta-hCG tests post-operatively.

## Discussion

Syncope, characterized by a transient loss of consciousness and postural tone, can be a manifestation of various underlying conditions, ranging from benign vasovagal episodes to life-threatening cardiovascular or neurological events. In women of reproductive age, the differential diagnosis of syncope must also include gynecologic and obstetric etiologies, particularly ruptured ectopic pregnancy and ruptured ovarian cysts. The incidence of syncope in the setting of ruptured ectopic pregnancy can vary widely; however, data specifically focusing on syncope as the primary presenting symptom without accompanying abdominal pain or vaginal bleeding are scarce.^1^,^2^ The prevalence of syncope associated with ruptured ovarian hemorrhagic cysts is similarly difficult to ascertain due to the frequent overlap with other symptoms such as abdominal pain and the possibility of asymptomatic occurrences.^5^

Distinguishing between ruptured ectopic pregnancy and ruptured ovarian cyst can be clinically challenging, especially when patients present with atypical symptoms. Given that clinical features of ovarian cyst complications are nonspecific, ultrasonography is critical.^6^ Transvaginal ultrasound is the preferred modality for evaluating ectopic pregnancy due to its superior resolution and ability to visualize early intrauterine pregnancies or adnexal masses.^7^ The sensitivity and specificity of transvaginal ultrasound for detecting ectopic pregnancies are approximately 88–90% and 97–99%, respectively.^8^,^9^ In cases of ruptured ectopic pregnancy, ultrasound may reveal a nonhomogeneous mass distinct from the ovary in 60% of ectopic pregnancy cases, but other hallmark signs like a hyperechoic ring, obvious gestational sac, and fetal pole are less common and depend on gestational age and ultrasound skill.^10^

Additionally, a ruptured ovarian ectopic pregnancy often cannot be definitively differentiated from a ruptured ovarian cyst via ultrasound because both conditions can manifest with similar findings such as intraperitoneal fluid and an adnexal mass.^10^ Our case underscores that although there are limitations of ultrasound in differentiating ectopic pregnancy from other gynecological conditions, it remains an important clinical tool when used in conjunction with lab work and clinical findings/presentations.

False-positive serum beta-hCG results can present significant diagnostic challenges. The incidence of false-positive pregnancy tests has not been closely studied or quantified well, but in general can vary depending on the population studied and the assay used. Several underlying mechanisms can contribute to false-positive beta-hCG results, including heterophile antibodies, phantom hCG, cross-reactivity with other hormones, and laboratory error.^11^,^12^,^13^

In the context of autoimmune disorders and immunosuppression, the risk of heterophile antibody interference may be increased. Heterophile antibodies are human antibodies that can bind to assay reagents, leading to falsely elevated hCG levels.^12^,^14^,^15^ This may be due to immune dysregulation and altered antibody production. In this case, the patient’s history of autoimmune hepatitis and immunosuppressant use may have contributed to the false-positive beta-hCG result. False positive beta-hCG results secondary to heterophile antibodies can also be associated with immunoglobulin A (IgA) deficiency, an important consideration to be aware of because 20–30% of individuals with IgA deficiency will have an autoimmune condition.^16^ One study showed that up to 30% of IgA deficient individuals tested had a false positive beta-hCG serum.^17^

This case emphasizes the importance of considering heterophile antibody interference as a potential cause of false-positive pregnancy tests, especially in patients with autoimmune disorders or those receiving immunosuppressive therapy. Further confirmatory testing, such as serial beta-hCG measurements and consideration of heterophile antibody blocking assays, may be warranted for accurate diagnosis; however, ruptured ectopic pregnancy is a time-sensitive emergency so emergent laparoscopy should not be delayed if there is clinical concern as exhibited in our case.^18^,^19^,^20^,^21^ For the emergency provider, it is valuable to understand that urine beta-hCG assays are generally less prone to interference than serum tests. The physiology underlying this is due to filtration of antibodies at the glomeruli, making heterophile antibody interference less likely in urine samples. As a result, a positive serum hCG with a negative urine hCG is often an indicator of heterophile antibody interference. In this patient’s case, repeat urine beta-hCG tests performed post-operatively confirmed the initial serum result was a false positive. This was most likely consistent with heterophile antibody interference given her history and demonstrates a valuable diagnostic tool that may help risk-stratify future similar patient presentations and workflows in the ED.

This case highlights the diagnostic challenges associated with evaluating women of reproductive age presenting with syncope and atypical symptoms. It is crucial to maintain a broad differential diagnosis and consider both obstetric and non-obstetric etiologies. While point-of-care ultrasound can be a valuable tool in the initial assessment, it has limitations in differentiating between ruptured ectopic pregnancy and ruptured ovarian hemorrhagic cysts, especially when clinical findings are equivocal. False-positive serum beta-hCG results, though rare and without statistical backing, have been reported due to a variety of factors including most commonly heterophile antibody interference.^13^,^17^ Ultimately, a comprehensive evaluation, including a detailed history, physical examination, and appropriate laboratory and imaging studies with a thorough understanding of the nuances of said tests in certain patient populations, is essential for accurate diagnosis and timely management of rare but can’t-miss emergencies in the ED. Depending on the clinical scenario and stability of the patient, consider confirming a positive serum beta-HCG with a urine HCG test in certain patient populations prior to invasive procedures and to potentially avoid exposure to unnecessary risk.

## Supplementary Information




















